# A cross-sectional electromyography assessment in linear scleroderma patients

**DOI:** 10.1186/1546-0096-12-27

**Published:** 2014-07-12

**Authors:** Claudia Saad Magalhães, Taciana de Albuquerque Pedrosa Fernandes, Thiago Dias Fernandes, Luis Antonio de Lima Resende

**Affiliations:** 1 Pediatric Rheumatology Unit, Pediatrics Division, Botucatu Medical School, São Paulo State University (UNESP), Botucatu, São Paulo, Brazil; 2 Electrophysiology Section, Neurology Division, Botucatu Medical School, São Paulo State University (UNESP), Botucatu, São Paulo, Brazil

**Keywords:** Electromyography, Linear scleroderma, Muscle, Peripheral nervous system

## Abstract

**Background:**

Muscle atrophy and asymmetric extremity growth is a common feature of linear scleroderma (LS). Extra-cutaneous features are also common and primary neurologic involvement, with sympathetic dysfunction, may have a pathogenic role in subcutaneous and muscle atrophy. The aim was investigate nerve conduction and muscle involvement by electromyography in pediatric patients with LS.

**Methods:**

We conducted a retrospective review of LS pediatric patients who had regular follow up at a single pediatric center from 1997–2013. We selected participants if they had consistently good follow up and enrolled consecutive patients in the study. We examined LS photos as well as clinical, serological and imaging findings. Electromyograms (EMG) were performed with bilateral symmetric technique, using surface and needle electrodes, comparing the affected side with the contralateral side. Abnormal muscle activity was categorized as a myopathic or neurogenic pattern.

**Results:**

Nine LS subjects were selected for EMG, 2 with Parry-Romberg/Hemifacial Atrophy Syndrome, 7 linear scleroderma of an extremity and 2 with mixed forms (linear and morphea). Electromyogram analysis indicated that all but one had asymmetric myopathic pattern in muscles underlying the linear streaks. Motor and sensory nerve conduction was also evaluated in upper and lower limbs and one presented a neurogenic pattern. Masticatory muscle testing showed a myopathic pattern in the atrophic face of 2 cases with head and face involvement.

**Conclusion:**

In our small series of LS patients, we found a surprising amount of muscle dysfunction by EMG. The muscle involvement may be possibly related to a secondary peripheral nerve involvement due to LS inflammation and fibrosis. Further collaborative studies to confirm these findings are needed.

## Background

Juvenile localized scleroderma represents the third most frequent rheumatic condition in children after juvenile idiopathic arthritis and systemic lupus erythematosus [1-3]. It comprises a group of conditions involving skin and subcutaneous tissues, resulting in fibrotic lesions with functional and cosmetic deformities [1,4-7]. There is no uniform terminology and classification, it was recently revised by an expert panel, defining the main presentations as circumscribed, generalized or pansclerotic morphea, linear scleroderma and mixed forms [8].

Linear scleroderma (LS) has a broad spectrum of clinical features. It can affect extremities, trunk, face and head. The term “*en coup de sabre*” is to further delineate linear scleroderma involving face and head. Facial lesions may progress involving muscle and underlying bones. Parry Romberg (PR) or Hemifacial atrophy (HFA) syndrome is characterized by tissue loss on one side of the face and some authors distinguish them as different entity due to degree of skin fibrosis [9-16]. The underlying pathogenesis remains unknown; however, it is associated with microvascular damage and altered collagen production, reported as a possible mechanism of injury.

Early diagnosis and effective treatment are crucial to improve the long-term outcome [1]. Muscle atrophy and asymmetric extremity growth is a common feature of LS [4,8]. Extra-cutaneous features are common findings [17-19] and primary neurologic involvement may have a pathogenic role [9,20]. It has been associated with central and peripheral nervous system involvement with facial palsy, extra-ocular movement disorders, trigeminal neuralgia and hemi-masticatory spams, all considered primary neurologic involvement [19,21,22] The etiology remains unclear, however autoimmunity, disturbed peripheral sympathetic nervous system, disturbed trigeminal nerve and early cerebral inflammation have all being proposed. Sympathetic regulation has pathogenic relevance in hemifacial atrophy (HFA). It was reproduced previously in our group by experimental sympathetic ganglion ablation in rabbits, cats and dogs [23]. Neurologic involvement includes complex partial seizures as well as abnormalities on computerized tomography (CT) and magnetic resonance imaging (MRI), reported as possible correlates to *“en coup de sabre,* HFA or PR syndrome [9].

There is still some discussion about classification of PR syndrome and HFA, the last representing the same features but without sclerosis at any stage [14-16]. Extremity linear streaks may follow a dermatome pattern distribution [24] and facial lesions were observed in association with Blaschko lines, that could be related to genetic mosaicism in the pattern of embryonic skin and neural cells migration [25].

Yet, there are few studies exploring nerve conduction and electromyography in those patients. Also, muscle involvement may contribute to extremity atrophy in linear and HFA forms. These neurological and muscular features of localized scleroderma led us to explore nerve conduction and muscle involvement by electromyography in LS.

## Methods

We evaluated a localized scleroderma series by retrospective chart review in a Pediatric Rheumatology Clinic, from 1997 to 2013.The diagnoses were made by one pediatric rheumatologist. Chart and pictures review was conducted by two pediatric rheumatologists. Consecutive cases seen in clinic during the last 18 months were included. There was no additional selection method other than clinic attendance during the study period. Parents and age-appropriated subjects signed informed consent. None who were invited refused to participate. This protocol was conducted according to Helsink Declaration. It was approved by the ethics committee from Botucatu Medical School – São Paulo State University (CEP-FMB-UNESP) under protocol number 441/2012.

Case-report forms characterizing features of skin and extra-cutaneous features, laboratory parameters, previous and current treatment, were completed by one pediatric rheumatologist. Diagnosis was made on clinical grounds describing lesions, skin thickness and pigmentation, skin and subcutaneous tissue atrophy, involvement of underlying muscle and bones, symmetry of face and extremities. Skin biopsy was performed in only 4 by the time active lesions were first identified. Active lesions were determined by the appearance of new lesions from the time of the last visit, observing signs of erythema, edema or increased size of a previous lesion. Pictures were also taken at the time of EMG exams. Neurologic exam was performed by the time of EMG test as a routine pediatric assessment of development, gross and fine movements, gait, tendon reflexes and functional assessment of cranial nerves by observing eye movements, smiling, chewing, hearing, swallowing, head turning and tongue protusion.

**Figure 1:**
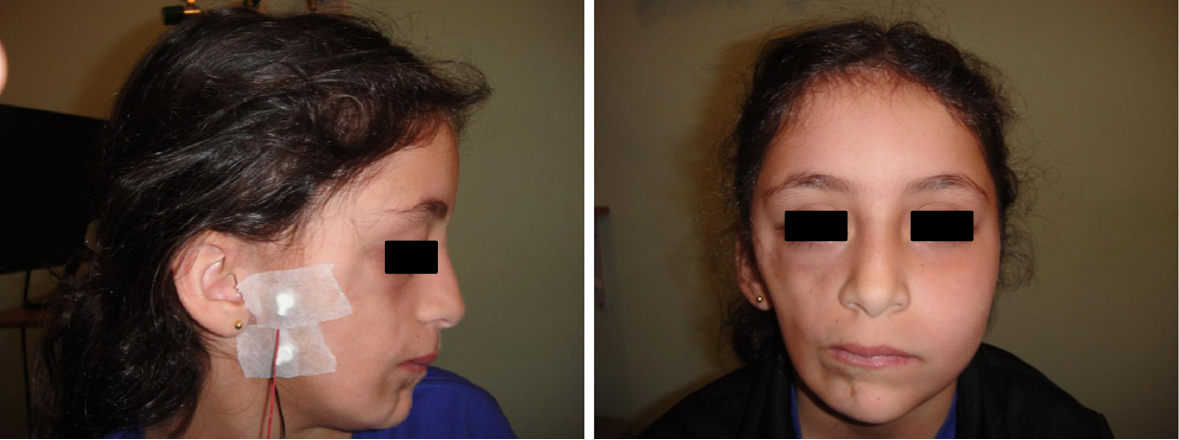
Placement of EMG surface electrodes in the face of a 7-year-old girl classified as Parry-Romberg syndrome.

Patients with face and head involvement were also investigated with brain CT, ophthalmologic and dental-maxillary assessments, without remarkable findings other than asymmetric face.Electromyography (EMG) was performed by 2 neurologists, using bilateral symmetric technique. EMG technique was carried out with axial needle electrodes for the first two patients. The surface electrodes technique became available and the following 7 cases had surface electrode EMG. It was performed at rest and during evoked effort in lying position. The electrodes were placed in the body area remarked by deep linear streaks over the limbs, trunk cliff-drop lesions or atrophic face side. The sensitivity was set to 20 μV/cm (at rest) or 200–1000 μV (during evoked effort), the analysis time was set to 10,200 or 500 ms/cm, and the filter band-pass to 20–10,000 Hz. All exams were performed in a Nihon-Kohden MEB 9400 equipment. Surface electrodes placement is presented in the Figure 1. Routine sensory and motor nerve conduction studies were performed with the same technique comparing affected side with the contralateral side.

EMG was categorized as normal or abnormal. The abnormal pattern was also categorized as myopathic and neurogenic patterns. Evaluation consisted on records of the presence or absence of spontaneous electric activity at rest, as positive sharp waves, fibrillation and fasciculation potentials, complex repetitive discharges. EMG at evoked effort consisted of evaluation of motor unit potentials, number of phases, duration, amplitude, morphology, stability from sequential and repetitive isolated motor units. Myopathic motor units have decreased duration and amplitude and neurogenic motor units have increased duration and amplitude. Degree of myopathic involvement was not measured by quantitative assessment.

## Results

Demographic and clinical characteristics at study entry are described in Table 1. There are some special cases. There was one female diagnosed in the first week of life, also called "congenital” LS [26]. Her mother had a recent diagnosis of systemic lupus erythematosus. A family history of autoimmune related diseases was also observed. Of note, during the study, the mother of one recently diagnosed LS case revealed for the first time similar lesions with childhood onset and she was referred to the adult specialist for confirmation of localized scleroderma. Eight patients were currently being treated or had previous treatment with methotrexate (15 mg per m^2^ of body surface). These eight patients had had also a short course of prednisone by the time of diagnosis [27,28]. None of these children had active lesions or were receiving prednisone by the time of EMG testing. All had documented clinical, serological and imaging findings of LS and normal routine neurologic examination.

**Table 1:** Demographic and clinical features of 9 patients with localized scleroderma

**Cases**	
Gender	
Male	2
Female	7
Age at onset (years)	
Mean	5.9
Median	3.7
Range	0 – 13.9
Diagnosis delay (months)	
Mean	6.3
Median	1.5
Range	0 – 25
Family history (number)	4/9
Scleroderma	1
SLE	1
Diabetes	5
Positive ANA*	3/8
Positive RF*	1/6

*8 tested 3 positive antinuclear antibodies, 6 tested , 1 positive rheumatoid factor.

Nine pediatric subjects, 7 girls, with LS were included in the EMG group. Overall 7 had LS on extremities, 2 had extremity and truncal lesions and 2 had face and head involvement. They were classified according to Padua Consensus classification [8]. Electromyogram analysis indicated that 8/9 children had a myopathic asymmetric pattern in muscles underlying extremity linear lesions. One had a neurogenic pattern. Motor and sensory nerve conduction study of upper and lower limbs resulted normal in 8/9, the one outlier was a patient with a deep linear streak in upper thigh. Masticatory muscle testing showed reduced root mean squares and increased turns per second in the atrophic face of two cases with HFA/PR Syndrome. These results are summarized in Table 2.

**Table 2:** Linear scleroderma classification, age, disease duration, affected body area, EMG pattern and technique

**Patient**	**Gender**	**Current Age (y)**	**Disease Duration at EMG test (y)**	**Localized Scleroderma Classification**	**EMG electrodes**	**EMG Pattern**	**Body area**
1	M	18	10	Linear	Needle	Neurogenic	Left thigh
2	F	17	2	Linear	Needle	Myopathic	Left arm
3	F	15	10	Mixed (trunk + limb linear + morphoea)	Surface	Myopathic	Right thigh
4	F	13	1	Mixed (trunk linear + morphoea)	Surface	Myopathic	Right dorsal trunk
5	F	8	8	Linear	Surface	Myopathic	Right forearm
6	F	16	15	Linear	Surface	Myopathic	Left thigh
7	M	13	3	Linear	Surface	Myopathic	Left forearm
8	F	16	9	HFA/PR	Surface	Myopathic	Left face
9	F	7	2	HFA/PR	Surface	Myopathic	Right face

HFA: hemifacial atrophy PR: Parry Romberg syndrome ; (y) years.

## Discussion

We developed the hypothesis that there might be muscle and nerve dysfunction in LS patients. In order to better define the role of peripheral nervous system and muscle involvement in all forms of LS, EMG seemed to be the most suitable technique for this study. We were able to show EMG myopathic patterns in 8/9 patients, thereby demonstrating abnormal electric activity in muscles underlying LS lesions. Interestingly, one patient had abnormal nerve conduction findings. We did also explore underlying muscle and nerve electric activity in patients seen in different phases of disease activity and progression of lesions. A wide variation of disease duration was observed. Unfortunately, these EMG techniques do not appear to distinguish well between active lesions and the sequelae due to fibrosis.

Previous studies exploring electrophysiology abnormalities in localized scleroderma are scarce case-reports in adults [29-33]. LS is indeed less frequent in adults [34,35] These studies have shown also abnormal electric activity in atrophic area indicating myopathic pattern [29,30,32]. There is one report of HFA with symptomatic masticatory spasms requiring treatment and trigeminal neuropathy was disclosed by nerve conduction studies [33]. Only one study in HFA affecting predominantly the tongue, failed to demonstrate neurogenic or myogenic process [31]. Neuroimaging was explored in a pediatric case of PR syndrome [9], speculating vasomotor disturbance and sympathetic dysfunction, but EMG was not performed. Seizures preceding face and scalp lesions were also described in another interesting pediatric report [36].

Myositis overlap at different degrees was also reported in localized scleroderma or associated connective tissue disease overlap features [29,37-39], but in these cases muscle weakness was present. None of our cases presented localized or generalized muscle weakness at any time of disease course.

Sympathetic dysfunction may play a pathogenic role in HFA/PR syndrome and this hypothesis has found some confirmation in our previous work because the ablation of the superior cervical ganglion in animal models reproduced clinical manifestations of HFA on the side of sympathectomy [23]. It is indeed possible that fat trophic changes of subcutaneous tissue is under influence of sympathetic nervous system and we hypothesized this could a common factor in different presentation of localized scleroderma.

There are limitations present in this study because of inherent nature of the LS disease. Our study was conducted during routine care, using a cross-sectional assessment of available cases. The availability of surface EMG electrodes techniques facilitated our study, as the needle insertion was a limiting factor for the assessment. Comparison of both techniques in adult patients in the same electrophysiology unit suggested that the two techniques of surface and inserted electrodes were comparable, but it was not possible to assess the degree of myopathic change with these techniques.

It is indeed challenging to distinguish disease activity and progression in LS, as markers of inflammation are usually of little value for diagnosis and follow up. Ultrasound imaging may provide clues to progression, but standardization for routine practice is still limited [40]. In practice, clinical exam of the lesions, sometimes leading to a confirmatory biopsy, is the usual way these LS are diagnosed and early treatment begun. Non standardized evaluation is the common practice for follow up, but a promising computerized tool has been developed to accurately define extension of the lesions and progression on follow up, but still not available on standard of care [41].

## Conclusion

Skin inflammation and fibrosis in LS, progressing to deep tissues with fat, muscle and bone atrophy need to be better elucidated. Nerve and muscle involvement in LS had not been previously investigated in pediatric patients. It is impossible to be sure if this abnormal findings were related to inflammation or fibrosis, so this preliminary observation needs further evaluation during different phases of disease activity using quantitative measure to assess the degree of myopathic and neurogenic changes.

## Competing interests

The authors declare that they have no competing interests.

## Authors’ contributions

CSM: conception; design; data acquisition, analysis, interpretation; manuscript writing and responsibility for accuracy and integrity of this work. TAPF: data acquisition, analysis, interpretation, manuscript approval. TDF: data acquisition, analysis and interpretation, manuscript approval. LALR: data acquisition, analysis and interpretation, manuscript approval. All authors read and approved the final manuscript.

## References

[B1] Zulian F Cuffaro G Sperotto F Scleroderma in children: an update Curr Opin Rheumatol 2013 25 5 643 650 23912318 10.1097/BOR.0b013e3283641f61

[B2] Herrick AL Ennis H Bhushan M Silman AJ Baildam EM Incidence of childhood linear scleroderma and systemic sclerosis in the UK and Ireland Arthritis Care Res (Hoboken) 2010 62 2 213 218 20191520 10.1002/acr.20070

[B3] Herrick AL Ennis H Bhushan M Silman AJ Baildam EM Clinical features of childhood localized scleroderma in an incidence cohort Rheumatology (Oxford) 2011 50 10 1865 1868 21729934 10.1093/rheumatology/ker142

[B4] Emery H Pediatric scleroderma Semin Cutan Med Surg 1998 17 1 41 47 9512106 10.1016/s1085-5629(98)80061-8

[B5] Zulian F Systemic sclerosis and localized scleroderma in childhood Rheum Dis Clin N Am 2008 34 1 239 255 ix 10.1016/j.rdc.2007.11.004 18329543

[B6] Torok KS Pediatric scleroderma: systemic or localized forms Pediatr Clin N Am 2012 59 2 381 405 10.1016/j.pcl.2012.03.011 PMC3459339 22560576

[B7] Chung L Lin J Furst DE Fiorentino D Systemic and localized scleroderma Clin Dermatol 2006 24 5 374 392 16966019 10.1016/j.clindermatol.2006.07.004

[B8] Laxer RM Zulian F Localized scleroderma Curr Opin Rheumatol 2006 18 6 606 613 17053506 10.1097/01.bor.0000245727.40630.c3

[B9] Cory RC Clayman DA Faillace WJ McKee SW Gama CH Clinical and radiologic findings in progressive facial hemiatrophy (Parry-Romberg syndrome) AJNR 1997 18 4 751 757 9127045 PMC8338508

[B10] Rai R Handa S Gupta S Kumar B Bilateral en coup de sabre-a rare entity Pediatr Dermatol 2000 17 3 222 224 10886757 10.1046/j.1525-1470.2000.01757.x

[B11] Orozco-Covarrubias L Guzman-Meza A Ridaura-Sanz C Carrasco Daza D Sosa-de-Martinez C Ruiz-Maldonado R Scleroderma 'en coup de sabre' and progressive facial hemiatrophy. Is it possible to differentiate them? J Eur Acad Dermatol Venereol 2002 16 4 361 366 12224693 10.1046/j.1468-3083.2002.00442.x

[B12] Korkmaz C Adapinar B Uysal S Beneficial effect of immunosuppressive drugs on Parry-Romberg syndrome: a case report and review of the literature South Med J 2005 98 9 940 942 16217992 10.1097/01.smj.0000177355.43001.ff

[B13] Sommer A Gambichler T Bacharach-Buhles M von Rothenburg T Altmeyer P Kreuter A Clinical and serological characteristics of progressive facial hemiatrophy: a case series of 12 patients J Am Acad Dermatol 2006 54 2 227 233 16443052 10.1016/j.jaad.2005.10.020

[B14] Kaliyadan F Biswas K Dharmaratnam AD Progressive facial hemiatrophy - a case series Indian J Dermatol 2011 56 5 557 560 22121278 10.4103/0019-5154.87155 PMC3221223

[B15] El-Kehdy J Abbas O Rubeiz N A review of Parry-Romberg syndrome J Am Acad Dermatol 2012 67 4 769 784 22405645 10.1016/j.jaad.2012.01.019

[B16] Tollefson MM Witman PM En coup de sabre morphea and Parry-Romberg syndrome: a retrospective review of 54 patients J Am Acad Dermatol 2007 56 2 257 263 17147965 10.1016/j.jaad.2006.10.959

[B17] Zulian F Athreya BH Laxer R Nelson AM de Oliveira SK F Punaro MG Cuttica R Higgins GC Van Suijlekom-Smit LW Moore TL Lindsley C Garcia-Consuegra J Esteves-Hilario MO Lepore L Silva CA Machado C Garay SM Uziel Y Martini G Foeldvari I Peserico A Woo P Harper J Juvenile localized scleroderma: clinical and epidemiological features in 750 children. An international study Rheumatology (Oxford) 2006 45 5 614 620 16368732 10.1093/rheumatology/kei251

[B18] Zulian F Vallongo C Woo P Russo R Ruperto N Harper J Espada G Corona F Mukamel M Vesely R Muzij-Nowakowska E Chaitow J Ros J Apaz MT Gerloni V Mazur-Zielinska H Nielsen S Ullman S Horneff G Wouters C Martini G Cimaz R Laxer R Athreya BH Localized scleroderma in childhood is not just a skin disease Arthritis Rheum 2005 52 9 2873 2881 16142730 10.1002/art.21264

[B19] Holland KE Steffes B Nocton JJ Schwabe MJ Jacobson RD Drolet BA Linear scleroderma en coup de sabre with associated neurologic abnormalities Pediatrics 2006 117 1 e132 e136 16326691 10.1542/peds.2005-0470

[B20] Lehman TJ The Parry Romberg syndrome of progressive facial hemiatrophy and linear scleroderma en coup de sabre. Mistaken diagnosis or overlapping conditions? J Rheumatol 1992 19 6 844 845 1404118

[B21] Amaral TN Marques Neto JF Lapa AT Peres FA Guirau CR Appenzeller S Neurologic involvement in scleroderma en coup de sabre Autoimmune Dis 2012 2012 719685 22319646 10.1155/2012/719685 PMC3272788

[B22] Amaral TN Peres FA Lapa AT Marques-Neto JF Appenzeller S Neurologic involvement in scleroderma: a systematic review Semin Arthritis Rheum 2013 43 3 335 347 23827688 10.1016/j.semarthrit.2013.05.002

[B23] Resende LA Dal Pai V Alves A Experimental study of progressive facial hemiatrophy: effects of cervical sympathectomy in animals Rev Neurol 1991 147 8–9 609 611 1962072

[B24] Jackson R Observations on the site, size, shape, and arrangement of lesions in the human skin Int J Dermatol 1984 23 6 370 375 6384080 10.1111/j.1365-4362.1984.tb03194.x

[B25] Soma Y Kawakami T Yamasaki E Sasaki R Mizoguchi M Linear scleroderma along Blaschko's lines in a patient with systematized morphea Acta Derm Venereol 2003 83 5 362 364 14609105 10.1080/00015550310013088

[B26] Zulian F Vallongo C de Oliveira SK Punaro MG Ros J Mazur-Zielinska H Galea P Da Dalt L Eichenfield LF Congenital localized scleroderma J Pediatr 2006 149 2 248 251 16887444 10.1016/j.jpeds.2006.04.052

[B27] Uziel Y Feldman BM Krafchik BR Yeung RS Laxer RM Methotrexate and corticosteroid therapy for pediatric localized scleroderma J Pediatr 2000 136 1 91 95 10636981 10.1016/s0022-3476(00)90056-8

[B28] Zulian F Martini G Vallongo C Vittadello F Falcini F Patrizi A Alessio M La Torre F Podda RA Gerloni V Cutrone M Belloni-Fortina A Paradisi M Martino S Perilongo G Methotrexate treatment in juvenile localized scleroderma: a randomized, double-blind, placebo-controlled trial Arthritis Rheum 2011 63 7 1998 2006 21305525 10.1002/art.30264

[B29] Stern LZ Payne CM Alvarez JT Hannapel LK Myopathy associated with linear scleroderma. A histochemical and electron microscopic study Neurology 1975 25 2 114 119 163451 10.1212/wnl.25.2.114

[B30] Parisi L Valente G Dell'Anna C Mariorenzi R Amabile G A case of facial hemiatrophy associated with linear scleroderma and homolateral masseter spasm Ital J Neurol Sci 1987 8 1 63 65 3570725 10.1007/BF02361438

[B31] Tan E Kurkcuoglu N Atalag M Gokoz A Zileli T Progressive hemifacial atrophy with localized scleroderma Eur Neurol 1989 29 1 15 17 2707287 10.1159/000116369

[B32] Malandrini A Dotti MT Federico A Selective ipsilateral neuromuscular involvement in a case of facial and somatic hemiatrophy Muscle Nerve 1997 20 7 890 892 9179164 10.1002/(sici)1097-4598(199707)20:7<890::aid-mus16>3.0.co;2-w

[B33] Kim HJ Jeon BS Lee KW Hemimasticatory spasm associated with localized scleroderma and facial hemiatrophy Arch Neurol 2000 57 4 576 580 10768634 10.1001/archneur.57.4.576

[B34] Atzeni F Bardoni A Cutolo M Hunzelmann N Krieg T Martini G Montecucco C Olski TM Secchi ME Valentini G Zulian F Sarzi-Puttini P Localized and systemic forms of scleroderma in adults and children Clin Exp Rheumatol 2006 24 1 Suppl 40 S36 S45 16466623

[B35] Marzano AV Menni S Parodi A Borghi A Fuligni A Fabbri P Caputo R Localized scleroderma in adults and children. Clinical and laboratory investigations on 239 cases Eur J Dent Educ 2003 13 2 171 176 12695134

[B36] Sartori S Martini G Calderone M Patrizi A Gobbi G Zulian F Severe epilepsy preceding by four months the onset of scleroderma en coup de sabre Clin Exp Rheumatol 2009 27 3 Suppl 54 64 67 19796565

[B37] Al Attia HM Ezzeddin H Khader T Aref MA A localised morphoea/idiopathic polymyositis overlap Clin Rheumatol 1996 15 3 307 309 8793268 10.1007/BF02229715

[B38] Voermans NC Pillen S de Jong EM Creemers MC Lammens M van Alfen N Morphea profunda presenting as a neuromuscular mimic J Clin Neuromuscul Dis 2008 9 4 407 414 18525425 10.1097/CND.0b013e318175c495

[B39] Ambade GR Dhurat RS Lade N Jerajani HR Childhood sclerodermatomyositis with generalized morphea Indian J Dermatol Venereol Leprol 2008 74 2 148 150 18388377 10.4103/0378-6323.39702

[B40] Li SC Liebling MS Ramji FG Opitz S Mohanta A Kornyat T Zhang S Dempsey-Robertson M Hamer C Edgerton S Jarrin J Malone M Doria AS Sonographic evaluation of pediatric localized scleroderma: preliminary disease assessment measures Pediatr Rheumatol Online J 2010 8 14 20423513 10.1186/1546-0096-8-14 PMC2878299

[B41] Zulian F Meneghesso D Grisan E Vittadello F Belloni Fortina A Pigozzi B Frigo AC Martini G Ruggeri A A new computerized method for the assessment of skin lesions in localized scleroderma Rheumatol (Oxford) 2007 46 5 856 860 10.1093/rheumatology/kel446 17264088

